# Predicting double-strand DNA breaks using epigenome marks or DNA at kilobase resolution

**DOI:** 10.1186/s13059-018-1411-7

**Published:** 2018-03-15

**Authors:** Raphaël Mourad, Krzysztof Ginalski, Gaëlle Legube, Olivier Cuvier

**Affiliations:** 1 LBME, Centre de Biologie Intégrative (CBI), Université de Toulouse, CNRS, UPS, 118, route de Narbonne, Toulouse, 31062 France; 2 Laboratory of Bioinformatics and Systems Biology, Centre of New Technologies, University of Warsaw, Zwirki i Wigury 93, Warsaw, 02-089 Poland; 3 LBCMCP, Centre de Biologie Intégrative (CBI), Université de Toulouse, CNRS, UPS, 118, route de Narbonne, Toulouse, 31062 France

**Keywords:** Double-strand breaks, Epigenetics, Chromatin, Machine learning

## Abstract

Double-strand breaks (DSBs) result from the attack of both DNA strands by multiple sources, including radiation and chemicals. DSBs can cause the abnormal chromosomal rearrangements associated with cancer. Recent techniques allow the genome-wide mapping of DSBs at high resolution, enabling the comprehensive study of their origins. However, these techniques are costly and challenging. Hence, we devise a computational approach to predict DSBs using the epigenomic and chromatin context, for which public data are readily available from the ENCODE project. We achieve excellent prediction accuracy at high resolution. We identify chromatin accessibility, activity, and long-range contacts as the best predictors.

**Electronic supplementary material:**

The online version of this article (10.1186/s13059-018-1411-7) contains supplementary material, which is available to authorized users.

## Background

Double-strand breaks (DSBs) arise when both DNA strands of the double helix are severed. DSBs are caused by the attack of deoxyribose and DNA bases by reactive oxygen species and other electrophilic molecules [1]. DSBs are particularly hazardous to a cell because they can lead to deletions, translocations, and fusions in the DNA, collectively referred to as chromosomal rearrangements [2]. DSBs are most commonly found in cancer cells. Several high-throughput sequencing techniques have been developed for the genome-wide mapping of DSBs in situ such as BLESS [3], GUIDE-seq [4], END-seq [5], and DSBCapture [6]. One of the most recent techniques, DSBCapture, was used to map more than 80 000 endogenous DSBs at a resolution lower than 1 kb in human. To date, DSBs have been mapped at high resolution only for a few cell lines due to the high sequencing costs and experimental difficulties. This has prevented the comprehensive study of the DSB landscape in the human genome across diverse cell lines and tissues.

Chromatin immunoprecipitation followed by high-throughput DNA sequencing (ChIP-seq) and DNase I hypersensitive site sequencing (DNase-seq) data are publicly available for dozens of cell lines and tissues from the ENCODE [7] and Roadmap Epigenomics [8] projects. On the one hand, recent studies have shown that the mapping of regulatory elements such as enhancers and promoters can be accurately predicted using available epigenome and chromatin data [9, 10]. Other studies have shown that the epigenome can be predicted by combinations of DNA motifs and DNA shape [11–14]. On the other hand, DSBs and the resulting DNA repair mechanisms have been shown to be linked to epigenome marks, including H3K4me1/2/3 and chromatin accessibility [6]. Accordingly, PRDM9-mediated trimethylation of H3K4 (H3K4me3) was originally shown to play a critical role in regulating DSBs associated with meiotic recombination hotspots [15–17]. Moreover, the repair of DSBs involves both post-translational modification of histones, in particular *γ*-H2AX, and concentration of DNA-repair proteins at the site of damage [18, 19]. It remains unclear to what extent DNA motifs or histone modifications predict or regulate the cellular response to DSBs in other developmental stages. Here, we thus sought to test whether publicly available epigenome and chromatin data, or DNA motifs and shape, could be used to predict DSBs.

In this article, we demonstrate, for the first time, that endogenous DSBs can be computationally predicted using the epigenomic and chromatin context, or using DNA sequence and DNA shape. Our predictions achieve excellent accuracy (area under the receiver operating characteristic curve or AUROC > 0.97) at high resolution (< 1 kb) using available ChIP-seq and DNase-seq data from public databases. Despite the highly imbalanced data when predicting DSBs genome-wide, our approach detects a reasonable number of false positives (area under the precision–recall curve or AUPR = 0.459). DNase, CTCF binding, and H3K4me1/2/3 are among the best predictors of DSBs, reflecting the importance of chromatin accessibility, activity, and long-range contacts in determining DSB sites and subsequent repairing. We also successfully predict DSB sites using DNA motif occurrences only (AUROC = 0.839) and identify the CTCF motif as a strong predictor. In addition, DNA shape analysis further reveals the importance of the structure-based readout in determining DSB sites, complementary to the sequence-based readout (motifs).

## Results and discussion

### Double-strand break prediction approach

**Fig. 1:**
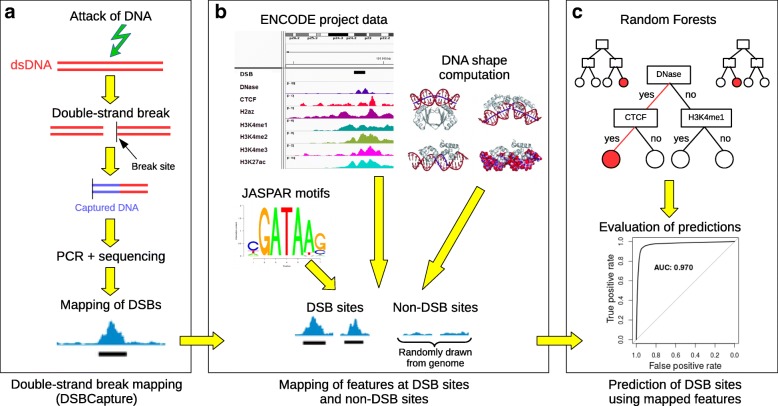
Double-strand break (DSB) prediction using epigenome marks or DNA. The prediction approach has three steps. **a** Mapping of DSBCapture sequencing data and DSB peak calling. **b** Mapping of features at DSB and non-DSB sites. Features include epigenomic and chromatin data from the ENCODE project, DNA motifs from the JASPAR database, and DNA shape predictions. **c** Prediction of DSB sites using features. AUC area under the curve, ds double strand, DSB double-strand break, PCR polymerase chain reaction

Our computational approach for predicting DSBs is schematically illustrated in Fig. 1. In the first step, we analyzed public DSBCapture data from Lensing el at. [6], which is the most sensitive and accurate genome-wide mapping of DSBs to date (Fig. 1a). DSBCapture captures DSBs in situ and it can directly map them at single-nucleotide resolution. DSBCapture peaks were called with less than 1-kb resolution (median size of 391 bases). The DSBCapture peaks obtained from two biological replicates were intersected to yield more reliable DSB sites. Endogenous breaks were captured for normal human epidermal keratinocytes (NHEKs), for which numerous ChIP-seq and DNase-seq data are publicly available from the ENCODE project [7]. In the second step, we integrated and mapped different types of data within DSB sites and non-DSB sites. To prevent bias effects, non-DSB sites were randomly drawn from the human genome with sizes, GC, and repeat contents similar to those of DSB sites [20] (Fig. 1b). ChIP-seq and DNase-seq peaks in NHEKs, as obtained from the ENCODE project, were mapped to corresponding DSB and non-DSB sites [7]. We also mapped p63 ChIP-seq peaks from keratinocytes [21]. We further searched for potential protein-binding sites at DSB and non-DSB sites using motif position weight matrices from the JASPAR 2016 database [22], and predicted DNA shape at DSB and non-DSB sites using Monte Carlo simulations [23]. In the third step, a random forest classifier was built to discriminate between DSB sites and non-DSB sites based on epigenome marks or DNA (Fig. 1c). Random forest variable importance values were used to estimate the predictive importance of a feature. We also compared random forest predictions with another popular method, lasso logistic regression [24]. Using lasso regression, we assessed the positive, negative, or null contribution of a feature to DSBs. We then split the DSB dataset into a training set to learn model parameters by cross-validation, and into a testing set to compute the receiver operating characteristic (ROC) and precision–recall (PR) curves, as well as AUROC and AUPR, to evaluate prediction accuracy.

### Double-strand breaks are enriched with epigenome marks and DNA motifs

**Fig. 2:**
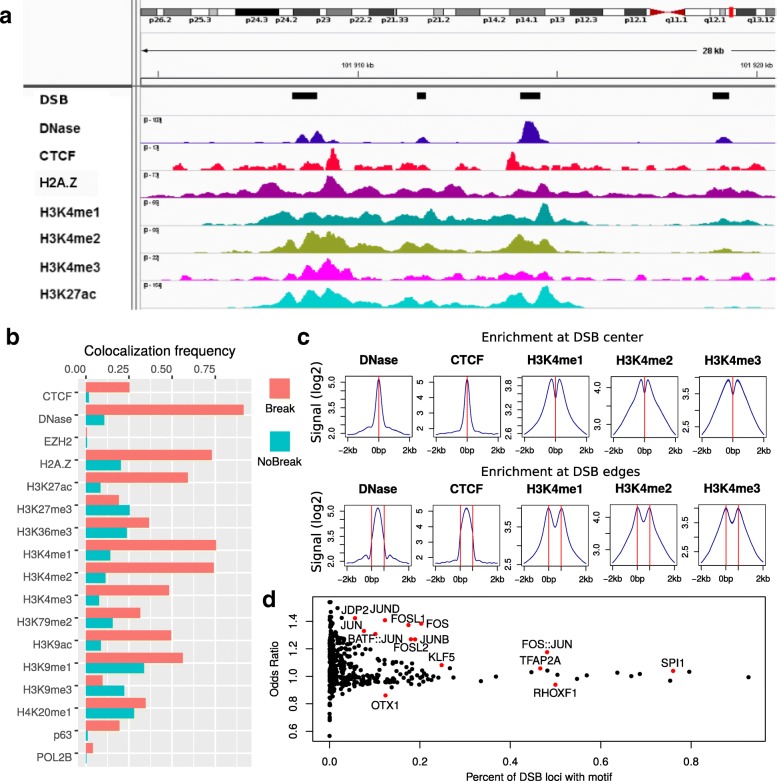
Epigenomic, chromatin, and DNA motif profiles of double-strand breaks (DSBs). **a** A genome browser view of DSBs with histone marks, chromatin openness (DNase-seq), and DNA-binding proteins. **b** Colocalization frequencies of epigenomic marks and DNA-binding proteins at DSB sites, compared to non-DSB sites. **c** Average profiles of epigenomic marks and DNA-binding proteins at DSB sites. **d** Enrichment of DNA motifs at DSB sites, as measured by the odds ratio and the percentage of DSB loci with a motif. DSB double-strand break

We first sought to assess comprehensively the link between DSBs and epigenome marks or DNA motifs. As previously shown [6, 25], several epigenomic and chromatin marks colocalized at DSBs (Fig. 2a). Among the most enriched marks were DNase I hypersensitive sites, H3H4 methylation, and CTCF (Fig. 2b). For instance, 91% of DSBs colocalized to a DNase site, whereas this percentage dropped to 11% for non-DSB regions. This corresponded to an odds ratio (OR) of 89.3. Similarly, high enrichment was found for H3K4me2 (74% versus 11%; OR=22.4) and for the insulator protein CTCF (25% versus 2%; OR=19), which may involve its interactions with the insulator-related cofactor cohesin, which has been shown to protect genes from DSBs [26]. As such, DSBs mostly localized within open and active regions that were often implicated in long-range contacts [27]. Interestingly, DSBs also colocalized with tumor protein p63 binding (19.4*%* versus 1%; OR=23.8), a member of the p53 gene family [28, 29]. In addition, we could distinguish DNase and CTCF sites that were enriched at the center of DSBs from histone marks that were found at the edges of DSB sites (Fig. 2c). Therefore, the strong enrichment of epigenomic and chromatin marks at DSB sites suggests that DSB regions could be accurately predicted using available ChIP-seq and DNase-seq data from public databases, including ENCODE and Roadmap Epigenomics.

Previous enrichment analyses of DNA-binding proteins were limited by the ChIP-seq data available. Hence, we sought DNA motifs that may be enriched at DSB sites as a way to obtain a more comprehensive list of candidate DNA-binding proteins. Of the 454 available motifs from the JASPAR 2016 database, 134 were significantly enriched (*p*<0.05, Bonferroni correction), indicating that DSBs were associated with a large number of protein-binding sites (Fig. 2d). Among the most enriched and frequent motifs, we identified numerous motifs specifically recognized by protein cofactors of the transcription factor complex AP-1. This included JUND (OR=1.40, 12% of DSBs), JUNB (OR=1.27, 19% of DSBs), the heterodimer BATF::JUN (OR=1.31, 10% of DSBs), and also FOS (OR=1.37, 20% of DSBs), FOSL1 (OR=1.37, 17% of DSBs), and FOSL2 (OR=1.27, 18% of DSBs). Among the most enriched but less frequent motifs, we expectedly found CTCF (OR=1.54, 1.7*%* of DSBs), as well as members of the tumor protein family p53, i.e., p53 itself (OR=1.54, 0.2*%* of DSBs), p63 (OR=1.49, 0.3*%* of DSBs), and p73 (OR=1.54, 0.1*%* of DSBs) [28, 29]. Such enrichment of DNA motifs at DSB sites, therefore, supports that DNA sequence can alone predict some of the DSBs encountered.

### Prediction using epigenomic and chromatin data

**Fig. 3:**
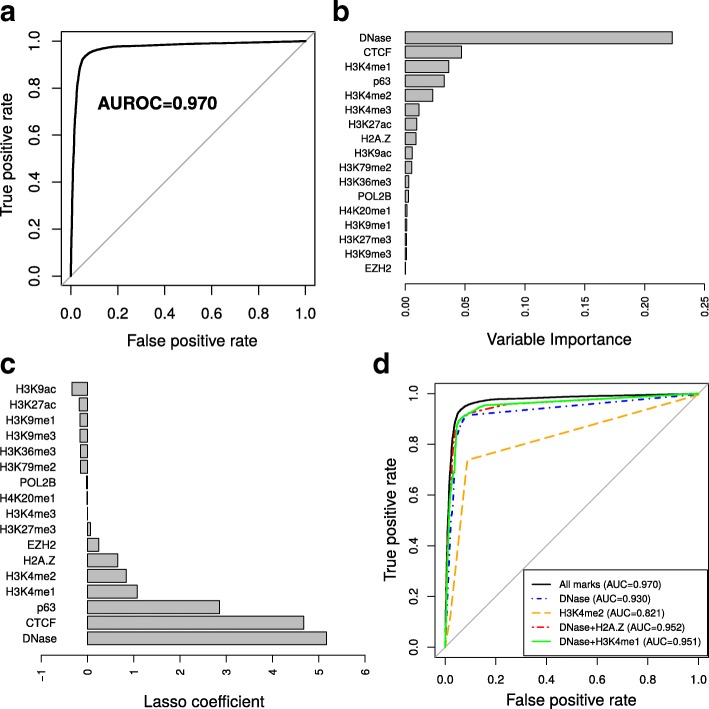
Prediction of double-strand breaks using epigenomic and chromatin data with random forests. **a** Receiver operating characteristic curve for the prediction of double-strand breaks. Area under the ROC curve (AUROC) is plotted. **b** Variable importance of epigenomic and chromatin variables. **c** Lasso logistic regression coefficients. **d** Different predictive models including all variables, DNase only, H3K4me2 only, DNase+H2A.Z, or DNase+H3K4me1. AUROC area under the receiver operating characteristic curve

Given the strong link between DSBs and epigenomic and chromatin marks, we sought to build a classifier to discriminate DSB sites from non-DSB sites based on the presence or absence of such marks. For this, we used random forests, which are very efficient classifiers for predicting a feature. They can capture non-linear and complex interaction effects [30]. We split the data into a training set to learn model parameters and a testing set to evaluate prediction accuracy. Using this classifier, we obtained excellent predictions of DSBs based on the epigenomic and chromatin marks available (AUROC = 0.970 and AUPR = 0.985; Fig. 3a; Additional file 1: Figure S1). Bootstrap analysis of 2000 replicates revealed that these predictions were very robust (95% confidence interval, CI, of AUROC: [0.968,0.972]). We also computed the variable importance (VI), which reflects the importance of a mark as a predictor (Fig. 3b). Among the marks, DNase showed the highest variable importance (VI=0.180), reflecting the known higher chromatin accessibility after DNA damage [19] or the involvement of chromatin-remodeling complexes in DSB processing [31]. Other good predictors were CTCF (VI=0.042), p63 (VI=0.031), H3K4me1 (VI=0.028), H3K4me2 (VI=0.019), H3K4me3 (VI=0.012), and H3K27ac (VI=0.010), highlighting the roles of active chromatin, but also long-range contacts and DNA damage response in predicting DSB sites.

A drawback of variable importance lies in its inability to distinguish between the positive or negative contribution of the predictive mark on DSBs. For this reason, we also used lasso logistic regression to predict DSBs [24]. With this second model, we obtained excellent predictions, although slightly less accurate (AUROC = 0.967, CI95*%*: [0.966,0.971]; AUPR = 0.982; Additional file 1: Figure S2). From lasso regression, we could assess the positive or negative contributions of the predictive marks using beta coefficients (Fig. 3c). We also performed logistic regression without any regularization and obtained very similar coefficients (Additional file 1: Figure S3). This allowed us to compute *p* values associated with the coefficients. We found that all variables, except H3K79me2, H3K9ac, and H4K20me1, were significantly associated with DSBs (Additional file 1: Table S1). We identified positive predictive contributions of DNase, CTCF, p63, H3K4me1, and H3K4me2 marks, as previously revealed by enrichment analysis. We also uncovered negative predictive contributions of H3K9ac, H3K36me3, and H3K79me2. In agreement, H3K9ac was shown to be rapidly and reversibly reduced in response to DNA damage [32]. Moreover, H3K36me3 may negatively impede DSBs by restricting chromatin accessibility through nucleosome positioning [33] or more directly by favoring the repair of DSBs [34].

We next sought to build a classifier using only one or two epigenomic marks, because this may be able to predict DSB sites even for cells for which only a few data points are available. We found that DNase I sites alone were sufficient to achieve good prediction accuracy (AUROC = 0.919 and AUPR = 0.962; Fig. 3d; Additional file 1: Figure S4), whereas H3K4me2 was not sufficient (AUROC = 0.816 and AUPR = 0.907; Fig. 3d; Additional file 1: Figure S4). Combinations of DNase with H2A.Z or H3K4me1 yielded very accurate predictions (AUROC = 0.952 and AUPR = 0.977; AUROC = 0.951 and AUPR = 0.976, respectively; Fig. 3d; Additional file 1: Figure S4), close to the model including all marks. Because DNase was a strong predictor, we explored where DNase was absent at DSBs to identify other marks that could be predictive here. We thus built a classifier using only DSBs that did not overlap any DNase site. DSB sites were still predicted well (AUROC = 0.869 and AUPR = 0.792; Additional file 1: Figure S5a and S5b), and CTCF and H3K4me1 were the most highly predictive variables (Additional file 1: Figure S5c). This revealed enhancer looping as a major driver of DSBs, in agreement with recent studies showing that DSBs form at loop anchors [35] and that CTCF facilitates DSB repair [36]. These results demonstrate that DSBs can be accurately predicted at less than 1-kb resolution using just a small amount of data.

### Comparison with BLESS experiment and validation using an independent dataset

**Fig. 4:**
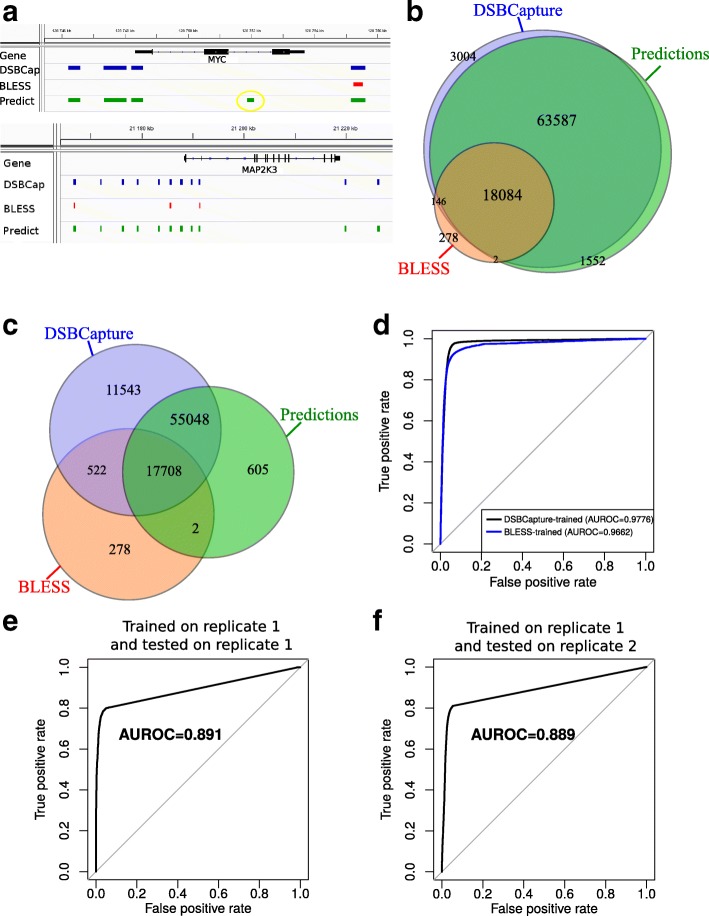
Comparison of predicted and BLESS double-strand breaks (DSBs) and validation with an independent dataset. **a** Comparison for the MYC and MAP2K3 genes. **b** Venn diagram illustrating the overlaps between DSBCapture, random forest DSBCapture-trained model predictions, and BLESS DSBs. **c** Venn diagram illustrating the overlaps between DSBCapture, random forest BLESS-trained model predictions, and BLESS DSBs. **d** Comparison of receiver operating characteristic (ROC) curves between DSBCapture-trained and BLESS-trained models. Areas under the ROC curves (AUROCs) are plotted. **e** ROC curve for the prediction of DSBs trained on replicate 1 and tested on the same replicate. **f** ROC curve for the prediction of DSBs trained on replicate 1 and tested on replicate 2. AUROC area under the ROC curve, DSB double-strand break, ROC receiver operating characteristic

We then compared previous DSB predictions with DSBs identified by BLESS experiments [3, 6]. We also included in the comparison DSBCapture DSBs as the gold standard because of its higher sensitivity compared to BLESS: 84 821 DSBs were found by DSBCapture compared to 18 510 DSBs found by BLESS [6]. We first looked at predicted DSB sites surrounding the two genes MYC and MAP2K3 (Fig. 4a). For MYC, random forests correctly identified the four DSBs that were detected by DSBCapture, but erroneously predicted one DSB (yellow circle), whereas BLESS identified only one DSB out of four. For MAP2K3, random forests successfully predicted all DSBs detected by DSBCapture, whereas BLESS identified only three DSBs out of 11.

We then compared predictions with BLESS at the genome-wide level (Fig. 4b). We observed that random forests correctly predicted 18 084 out of 18 510 DSB sites (97.70*%*) found by BLESS, while it also successfully identified an additional 63 587 out of 66 591 DSB sites (95.49*%*) found by DSBCapture that were not detected by BLESS. The model misclassified only 1552 out of 83 225 predicted DSB sites (1.86*%*). However, this previous prediction comparison should be carefully interpreted, because the model was learned from DSBCapture and then used to predict DSBCapture and BLESS DSBs.

To demonstrate the power of model-based predictions further, we devised another computational experiment, which consisted of training the model with BLESS DSBs and then predicting DSBCapture DSBs to test if the model could predict DSBCapture DSBs that were not detected by BLESS. Very interestingly, we found that the model was able to predict an additional 55 048 out of 84 821 DSBs (64.90*%*) that were detected by DSBCapture but not by BLESS, and it identified only 605 DSBs out of 73 363 predicted DSBs (0.82*%*), which may be false positives not detected by DSBCapture and BLESS (Fig. 4c).

We then sought to compare models learned using DSBCapture and BLESS DSBs with a fair benchmark. For this, we devised the following strategy. A first model was learned from DSBCapture and was used to predict BLESS DSB sites (the DSBCapture-trained model), and a second model was learned from BLESS and was used to predict DSBCapture DSB sites (the BLESS-trained model). We found that both models had very good prediction performance (AUROCmodel1=0.9776 and AUPRmodel1=0.971; AUROCmodel2=0.9662 and AUPRmodel2=0.983; Fig. 4d; Additional file 1: Figure S6).

In the previous section, we evaluated the accuracy of model predictions using a testing dataset that was from the same data as the training data (DSBs that overlapped between two replicates were split into a training dataset and a testing dataset). Here, we assessed model predictions by training random forests on one biological replicate and by testing prediction accuracy on a second biological replicate. For this, we used the two available DSBCapture biological replicates [6]. Accordingly, we used ENCODE epigenomic and chromatin data for which two biological replicates were available: DNase, CTCF, H3K4me3, H3K27me3, and H3K36me3. The first (respectively, second) replicates of the ENCODE data were associated with the first (respectively, second) DSBCapture replicate. Using only those five DNase-seq and ChIP-seq items, the model that was learned with the first replicate achieved accurate predictions on the testing data from the first replicate (AUROC = 0.891 and AUPR = 0.906; Fig. 4e; Additional file 1: Figure S7a). Note that the observed lower accuracy compared to that in the previous section (Fig. 3a,d) can be explained by the small amount of available epigenomic and chromatin data, and the lower reliability of DSBs identified using only one DSBCapture replicate. To validate the model on an independent dataset, we predicted DSBs from the second replicate using the model trained on the first replicate together with DNase-seq and ChIP-seq data for the second replicate. We obtained accurate predictions close to that obtained for the first replicate (AUROC = 0.889 and AUPR = 0.913; Fig. 4f; Additional file 1: Figure S7b). These accurate predictions demonstrate that using a classifier trained with epigenome and chromatin data is a reliable strategy for predicting DSBs.

### The impact of controls on prediction

**Fig. 5:**
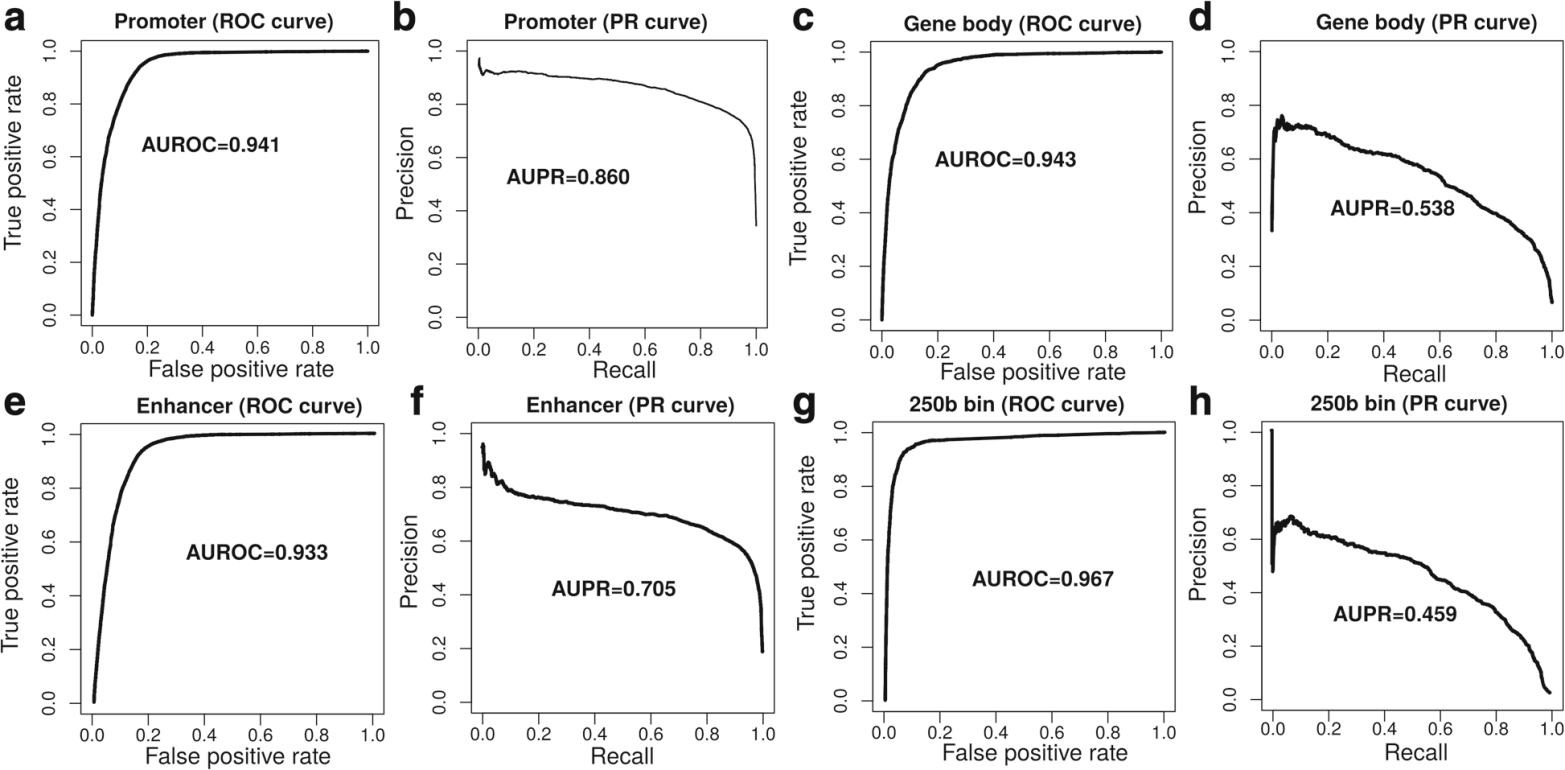
Prediction of double-strand breaks (DSBs) using different controls. **a** Receiver operating characteristic (ROC) curve of a random forest discriminating between promoters with DSBs and promoters without. Area under the ROC curve (AUROC) is plotted. **b** Precision–recall (PR) curve of the random forest used in (**a**). Area under the PR curve (AUPR) is plotted. **c** ROC curve of a random forest discriminating between gene bodies with DSBs and gene bodies without. **d** Precision–recall curve of the random forest used in (**c**). **e** ROC curve of a random forest discriminating between enhancers with DSBs and enhancers without. **f** Precision–recall curve of the random forest used in (**e**). **g** ROC curve of a random forest discriminating between 250-base bins with DSBs and 250-base bins without. **h** Precision–recall curve of the random forest used in (**g**). AUPR, area under the PR curve, AUROC area under the ROC curve, DSB double-strand break, PR precision–recall, ROC receiver operating characteristic

To assess if the high predictive accuracy of the model was inflated due to the way we selected non-DSB sites (the negative class), we devised different strategies. We first focused on gene promoters and built a random forest classifier to discriminate between promoters with DSBs (16 801 sites) and promoters without (48 838 sites). As previously done, we computed the ROC curve but we also included the PR curve to account for class imbalance. We obtained very good performance for both the ROC curve (AUROC = 0.941; Fig. 5a) and the PR curve (AUPR = 0.860; Fig. 5b). Second, we built a classifier to discriminate between gene bodies with DSBs (2187 sites) and gene bodies without (34 573 sites). We also obtained a very good ROC curve (AUROC = 0.943; Fig. 5c), but with a lower PR curve because of the higher class imbalance in gene bodies (AUPR = 0.538; Fig. 5d). Third, we built a classifier to discriminate between enhancers with DSBs (7373 sites) and enhancers without (38 521 sites). We again observed a very good ROC curve (AUROC = 0.933; Fig. 5e) and good PR (AUPR = 0.705; Fig. 5f). Fourth, we evaluated predictions over the whole genome in an unbiased way. For this, we split the genome into 250-base bins. Then we built a classifier to discriminate between bins with DSBs (189 132 bins) and bins without (11 362 262 bins). Using this approach, we obtained very good ROC accuracy (AUROC = 0.967) but with lower PR accuracy (AUPR = 0.459) due to the high class imbalance, revealing a high number of false positives detected genome-wide by our method. We concluded that the excellent accuracy of model-based predictions was not inflated due to the way non-DSB sites were selected over the genome.

### Prediction in another cell type

To validate our model-based predictions further, we used the random forest learned from DSBs in one cell type (NHEK) to predict DSBs in another cell type (U2OS). For this, we used data that were available for both NHEK and U2OS cells: DNA-seq, CTCF, H3K4me1/3, H3K9me3, H3K27ac, H3K27me3, H3K36me3, and POL2B. The validation is illustrated in Additional file 1: Figure S8. In summary, we trained a random forest with DSBCapture DSBs and DNase-seq and ChIP-seq data in NHEKs. We then predicted DSBs in U2OS cells using the NHEK-trained random forest with U2OS DNA-seq and ChIP-seq data. We validated the predictions with U2OS DSB data.

To evaluate prediction accuracy, we used the DSB data (DSBCapture [6] and BLESS [37]) that were generated for a specific cell line called U20S AID-DIvA. These DSB data were the only ones available in U20S. This cell line was a U2OS cell line that expressed the AsiSI restriction enzyme inducing DSBs at targeted sites [38]. To focus on endogenous DSBs, we kept only DSB data that did not overlap AsiSI sites. Most likely, only a fraction of all endogenous DSBs in U2OS could be mapped because DSB read coverage was low outside AsiSI sites.

**Fig. 6:**
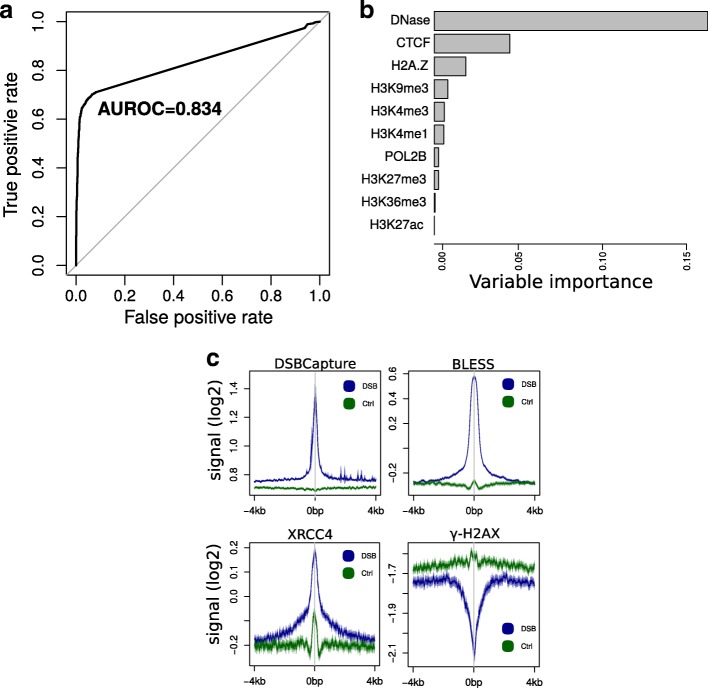
Prediction of double-strand breaks (DSBs) using a random forest learned from DSBs in one cell type (NHEK) to predict DSBs in another cell type (U2OS). **a** Receiver operating characteristic (ROC) curve to predict U2OS DSBs using the NHEK-learned random forest. Area under the ROC curve (AUROC) is plotted. **b** Variable importance from the prediction of U2OS DSBs using the U2OS-learned random forest. **c** Average profiles of DSBCapture, BLESS, XRCC4, and *γ*-H2AX at predicted DSB regions compared to non-DSB regions over the whole genome. AUROC area under the ROC curve, DSB double-strand break, ROC receiver operating characteristic

In the first benchmark, we computed ROC and PR curves to evaluate the accuracy of model-based predictions. We compared our DSB predictions to a list of 2327 DSB sites identified by DSBCapture peak calling and 6443 non-DSB sites that were randomly drawn. Although this endogenous DSB list was far from complete, we obtained good prediction accuracy (AUROC = 0.835; CI95*%*: [0.824,0.846]; AUPR = 0.881; Fig. 6a; Additional file 1: Figure. S9). In agreement, we found that U2OS DSB prediction using a U2OS-trained random forest yielded only slightly better predictions than using a NHEK-trained random forest (AUROC = 0.859; CI95*%*: [0.849,0.868]; AUPR = 0.904; Additional file 1: Figure S10). Moreover, DNase and CTCF had the highest variable importance, as found in NHEKs (Fig. 6b). Unfortunately, we could not carry out the same ROC and PR curve analyses with the BLESS data because not enough DSB sites were identified by peak calling.

In the second benchmark, we split the genome into 250-base bins and then predicted DSBs genome-wide. The model identified 87 190 bins with a high DSB score (predicted DSBs) and 77 510 bins with a low DSB score (predicted controls). As expected, we found a high enrichment of both DSBCapture and BLESS reads at predicted DSBs compared to predicted controls (Fig. 6c). On average, both DSBCapture and BLESS signals accordingly increased with the predicted DSB signal (Additional file 1: Figure S11a,b). Fortunately, there were also ChIP-seq data available for XRCC4, a DNA repair protein involved in non-homologous end-joining. Hence, we looked at whether XRCC4 was recruited at predicted DSBs. We found a high enrichment of XRCC4 at predicted DSBs compared to predicted controls (Fig. 6c), and an increase of the XRCC4 signal depending on the predicted DSB signal (Additional file 1: Figure S11c). In addition, ChIP-seq data were available for *γ*-H2AX, a histone mark that is induced at a megabase domain scale after DSBs, but is depleted on the few kilobases surrounding the exact break point [38, 39]. Accordingly, we observed that *γ*-H2AX was depleted at predicted DSBs compared to predicted controls (Fig. 6c), and we found a decrease of the *γ*-H2AX signal with the predicted DSB signal (Additional file 1: Figure S11d).

Additionally, we performed genome-wide DSB predictions in two other cell types for which endogenous DSB data were available, namely KBM7 (chronic myelogenous leukemia) and MCF-7 (breast cancer). For KBM7 cells, we used DNase-seq, CTCF, H3K4me1/me3, and H3K9me3 for prediction and BLISS for validation [40]. The model identified 163 113 bins with a high DSB score (predicted DSBs) and 115 204 bins with a low DSB score (predicted controls). We found an enrichment of BLISS reads at predicted DSBs compared to predicted controls (Additional file 1: Figure S12a). On average, the BLISS signal accordingly increased with the predicted DSB signal (Additional file 1: Figure S12b). For MCF-7 cells, we used DNase-seq, CTCF, H3K4me1/me3, H3K9ac/me3, and H3K27me3 for prediction and END-seq for validation [35]. The model identified 54 746 bins with a high DSB score (predicted DSBs) and 84 576 bins with a low DSB score (predicted controls). As expected, we found an enrichment of END-seq reads at predicted DSBs compared to predicted controls (Additional file 1: Figure S12c). On average, the END-seq signal accordingly increased with the predicted DSB signal (Additional file 1: Figure S12d). We also tested whether our predictions in MCF-7 cells overlapped etoposide (ETO) induced DSBs mapped by END-seq. Interestingly, we found a strong enrichment of ETO END-seq reads at predicted DSBs compared to predicted controls (Additional file 1: Figure S12e). On average, the END-seq signal accordingly increased with the predicted DSB signal (Additional file 1: Figure S12f).

All these results revealed that the strongest predictors including DNase and CTCF were the same in two different cell types, and that accordingly, a random forest learned in one cell type can efficiently predict DSBs in another cell type.

### Prediction from DNA motifs and shape

**Fig. 7:**
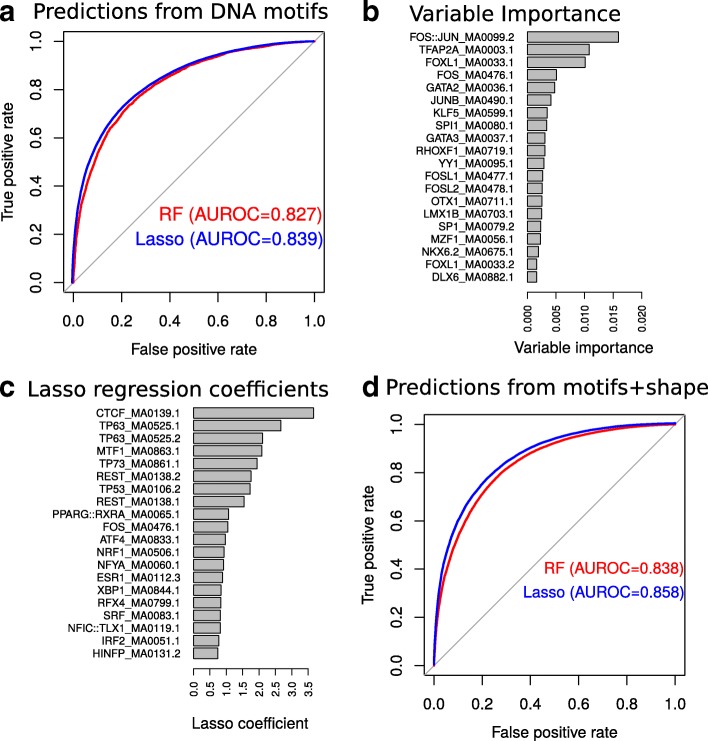
Prediction of double-strand breaks (DSBs) using DNA motifs and shape. **a** Receiver operating characteristic (ROC) curve for the DSB predictions using DNA motifs from the JASPAR 2016 database. Random forest (RF) and lasso logistic regression were compared. **b** The 20 highest DNA motif variable importance values. **c** The 20 highest DNA motif lasso coefficients. **d** ROC curve for the DSB predictions using DNA motifs with DNA shape. AUROC area under the ROC curve, DSB double-strand break, RF random forest, ROC receiver operating characteristic

We then explored the possibility of predicting DSBs based on DNA sequence using DNA motif occurrences. We built a random forest classifier using 454 available motifs from the JASPAR 2016 database and obtained good prediction accuracy (AUROC = 0.827; CI95*%*: [0.819,0.831]; AUPR = 0.910; Fig. 7a; Additional file 1: Figure S13a). Several motifs from the transcription factor complex AP-1 were good predictors, such as FOS::JUN (VI=0.016) and FOS (VI=0.009) (Fig. 7b), which were previously shown to be enriched at DSB sites (see Section “Results and discussion”, DSBs are enriched with epigenome marks and DNA motifs). Using lasso regression, we improved previous predictions (AUROC = 0.839; CI95*%*: [0.829,0.840]; AUPR = 0.919; Fig. 7a; Additional file 1: Figure S13a). Based on lasso regression, we found that the CTCF motif had the highest beta coefficient (*β*=3.22), corresponding to OR=25 (Fig. 7c), supporting recent evidence showing that long-range contacts are involved in DNA repair [25, 35, 41]. Furthermore, motifs of tumor proteins p53, p63, and p73 had high coefficients (*β*>2.03, OR>7.6), in agreement with previous predictions based on ChIP-seq data (see above). We also found motifs recognized by factors involved in heavy metal response (MTF-1: *β*=2.08, OR=8), in oxidative stress response (NRF1: *β*=0.93, OR=2.53; REST: *β*=1.75, OR=5.75), in endoplasmic reticulum stress (ATF4: *β*=0.97, OR=2.64), and in estrogen-induced DNA damage (ESR1: *β*=0.88, OR=2.41). To assess the significance of those motifs, we built a logistic regression model without any regularization including all motifs with *β*>0.5. We found that most motifs (22/29) were significantly associated with DSBs (*p*<0.05 after false discovery correction; Additional file 1: Table S2). Many of the above mentioned proteins have been shown to interact with each other. For instance, NRF1 associates with Jun proteins of the AP-1 complex [42]. ESR1 associates with AP-1/JUN and FOS to mediate estrogen element response-independent signaling [43].

DNA shape was recently shown to predict transcription factor binding sites and gene expression [14, 44]. Thus, we assessed if DNA shape could similarly serve to predict DSBs together with motifs. For this, we predicted four DNA shape features using simulations: minor groove width (MGW), propeller twist (ProT), roll (Roll), and helix twist (HelT) of DSB sites at base resolution. From each feature, we computed 12 predictors including quantiles (0, 10, 20, 30, 40, 50, 60, 70, 80, 90, and 100%) and the variance to describe the distribution of the feature within a DSB site. We used the resulting 48 variables combined with motif occurrences to predict DSBs with random forests and obtained better accuracy (AUROC = 0.838 and AUPR = 0.915; Fig. 7d; Additional file 1: Figure S13b) compared to using motifs alone (AUROC = 0.827 and AUPR = 0.910; Fig. 7a; Additional file 1: Figure S13a). Among the DNA shape variables, ProT median and MGW variance had the highest variable importance (VI=0.01 and VI=0.01, respectively). Using lasso regression, we also obtained better predictions (AUROC = 0.858), compared to using motifs only (AUROC = 0.839 and AUPR = 0.928; Fig. 7d; Additional file 1: Figure S13b). These results reflect the importance of DNA shape in determining DSB sites, in agreement with studies showing that narrow minor grooves (created by either sequence context or DNA bending) limit access of reactive oxygen species [45].

## Conclusions

DSBs are a major threat to a cell and they are associated with cancer development. Over the past years, new techniques have been developed to map DSBs at high resolution and genome-wide level. However, these techniques are costly and challenging. Here, we show, for the first time, that such DSBs can be computationally predicted using public epigenomic data, even when the availability of data is limited (e.g., DNase I and H3K4me1). By using state-of-the-art computational models, we achieve excellent prediction accuracy, paving the way for a better understanding of DSB formation depending on developmental stage or cell-type specific epigenetic marks. Thus, our computational approach should allow the genome-wide mapping of DSBs in numerous cell lines and tissues using the ENCODE and Roadmap Epigenomics databases.

There are multiple perspectives for this work. Recent developments from deep (convolutional) neural networks [13, 46] can improve model predictions and decrease the number of false positives at the genome level. In addition, our current model did not account for the impact of copy number variation in cancer cells on prediction, and future studies should integrate copy number variation as a quantitative predictor variable in the model to correct for this bias.

## Methods

### Double-strand breaks

All double-strand DNA break data used are summarized in Table 1. We used double-strand DNA breaks mapped by DSBCapture and BLESS in human epidermal keratinocyte (NHEK) cells from the Gene Expression Omnibus (GEO) accession GSE78172 [6]. DSBCapture and BLESS peaks were called using MACS 2.1.0 on human genome assembly hg19 (https://github.com/taoliu/MACS). The peaks obtained from two biological replicates were intersected to yield more reliable DSB sites for model predictions.

**Table 1:** Double-strand DNA break data summary

Cell line	Treatment	Technique	Number of replicates	Accession
NHEK	No treatment	DSBCapture	2	GSE78172
NHEK	No treatment	BLESS	2	GSE78172
U2OS	4-hydroxytamoxifen	DSBCapture	1	GSE78172
U2OS	No treatment	BLESS	1	E-MTAB-4846
KBM7	No treatment	BLISS	1	SRP099132
MCF-7	No treatment	END-seq	1	GSE99197
MCF-7	Etoposide	END-seq	1	GSE99197

We used double-strand DNA breaks mapped by DSBCapture and BLESS in AID-DIvA cells, a U2OS cell line (human bone osteosarcoma epithelial cells) expressing the AsiSI restriction enzyme fused to a modified estrogen receptor ligand-binding domain [38]. Upon tamoxifen treatment, AsiSI induces sequence-specific DSBs at GCGATCGC sites. DSBCapture data were from tamoxifen-treated cells from GEO accession GSE78172 [6]. DSBCapture peaks were called using MACS 2.1.0 on human genome assembly hg19. BLESS data were from untreated cells arrested in G1 phase from ArrayExpress accession E-MTAB-4846 [37]. Because of the low coverage of BLESS data, a sufficient number of DSB peaks could not be called.

We used double-strand DNA breaks mapped by BLISS in KBM7 cells (human myeloid leukemia) from NCBI Sequence Read Archive at SRP099132 [40]. We also used double-strand DNA breaks mapped by END-seq in untreated and etoposide-treated MCF-7 cells (human breast cancer) from GSE99197 [35].

### ChIP-seq and DNase-seq data

All ChIP-seq and DNase-seq data used are summarized in Table 2. We used ChIP-seq uniform peaks (CTCF, POL2B, EZH2, H3K4me1/me2/me3, H3K9me1/me3/ac, H3K27me3/ac, H3K36me3, H3K79me2, H4K20me1, and H2A.Z) and DNase-seq uniform peaks for NHEKs from the ENCODE project [7] (https://genome.ucsc.edu/encode). We also used p63 ChIP-seq of keratinocytes from GEO accession GSE59827 [21].

**Table 2:** ChIP-seq and DNase-seq data summary

Cell line	Treatment	Technique	Number of replicates	Accession
NHEK	No treatment	CTCF, H3K4me3, H3K27me3, H3K36me3 ChIP-seq	2	ENCODE uniform peaks
NHEK	No treatment	EZH2, H3K4me1/me2, H3K9me1/me3/ac, H3K79me2, H4K20me1, H2A.Z, H3K27ac, POL2B ChIP-seq	1	ENCODE uniform peaks
NHEK	No treatment	DNase-seq	2	ENCODE uniform peaks
NHEK	No treatment	p63 ChIP-seq	1	GSE59827
U2OS	No treatment	DNase-seq, H3K27ac ChIP-seq	1	GSE87831
U2OS	No treatment	H3K4me1, POL2B ChIP-seq	1	GSE73742
U2OS	No treatment	H3K4me3, H3K27me3 ChIP-seq	1	GSE35573
U2OS	No treatment	H3K9me3, H3K36me3 ChIP-seq	1	ENCODE
U2OS	No treatment	CTCF ChIP-seq	1	ChIP-Atlas
U2OS	4-hydroxytamoxifen	XRCC4, *γ*-H2A.X ChIP-seq	1	E-MTAB-1241
KBM7	No treatment	DNase-seq	1	ChIP-Atlas
KBM7	No treatment	H3K9me3 ChIP-seq	1	GSE60056
K562	No treatment	CTCF, H3K4me1/me3 ChIP-seq	1	ENCODE
MCF-7	No treatment	H3K4me1/me3, H3K9ac/me3, H3K27me3 ChIP-seq	1	GSE23701
MCF-7	No treatment	DNase-seq and CTCF ChIP-seq	1	ENCODE

For U2OS cells, we used DNase-seq and H3K27ac ChIP-seq peaks from GEO accession GSE87831 [47]. We used H3K4me1 and POL2B ChIP-seq peaks from GEO accession GSE73742 [48]. We used H3K4me3 and H3K27me3 ChIP-seq peaks from GSE35573 [49]. We used H3K9me3 and H3K36me3 ChIP-seq peaks from ENCODE [7]. We used CTCF ChIP-seq peaks from the ChIP-Atlas database (http://chip-atlas.org/). We used XRCC4 and *γ*-H2A.X ChIP-seq for tamoxifen-treated DIvA cells from ArrayExpress accession E-MTAB-1241 [37].

For KBM7 cells, we used DNase-seq from the ChIP-Atlas database, and H3K9me3 ChIP-seq from GSE60056 [50]. Instead of KBM7, we used K562 (chronic myelogenous leukemia) for CTCF, H3K4me1/me3 ChIP-seq from the ENCODE project [7] (https://genome.ucsc.edu/encode). For MCF-7 cells, we used H3K4me1/me3, H3K9ac/me3, and H3K27me3 ChIP-seq without treatment (DMSO) from GSE23701 [51, 52]. We used DNase-seq and CTCF ChIP-seq from ENCODE [7].

### DNA motifs

We used motif position frequency matrices for transcription factor binding sites from the JASPAR 2016 database (http://jaspar.genereg.net). We called transcription factor binding sites over the human genome using the position weight matrices and a minimum matching score of 80%.

### DNA shape

We predicted four DNA shape features using Monte Carlo simulations: minor groove width (MGW) and propeller twist (ProT) at base pair resolution and roll (Roll) and helix twist (HelT) at base pair step resolution using R package DNAshapeR (https://bioconductor.org/packages/release/bioc/html/DNAshapeR.html).

### Random forest and lasso regression

We used R package ranger (https://cran.r-project.org/web/packages/ranger) to compute the random forest classification efficiently [30]. We used the default package parameters: num.trees=500 and mtry is the square root of the number of variables. Variable importance was computed using the mean decrease in accuracy in the out-of-bag sample. To discriminate between DSB and non-DSB sites, we randomly selected genomic sequences that matched sizes, GC, and repeat contents of DSB sites using R package gkmSVM (https://cran.r-project.org/web/packages/gkmSVM). To learn the model, we mapped epigenomic data, DNA motifs, and DNA shape as follows. For epigenomic data including ChIP-seq and DNase-seq data, we used peak genomic coordinates of a feature (for instance, CTCF binding sites) and considered the presence (*x*=1) or absence (*x*=0) of the corresponding feature at the DSB site. If a feature peak overlapped only 60% of the DSB site, then *x*=0.6. For DNA motifs, we computed the number of motif occurrences within DSB and non-DSB sites. For DNA shape, we computed four features including MGW, ProT, Roll, and HelT of DSB sites at base resolution. For each DNA shape feature, we then computed 12 predictors, including quantiles (0, 10, 20, 30, 40, 50, 60, 70, 80, 90, and 100%) and the variance to describe the distribution of the feature within a DSB site. The DSB data were next split into two sets: the training set used for learning the model and a test set used for assessing prediction accuracy. We also used R package glmnet (https://cran.r-project.org/web/packages/glmnet/index.html) to compute lasso logistic regression with cross-validation. To assess the prediction accuracy of random forest and lasso regression, we computed the ROC curve and AUROC. To estimate the confidence interval for AUROC, we used the pROC R package (https://cran.r-project.org/web/packages/pROC). We also computed the PR curve and AUPR to assess prediction accuracy when the classes were very imbalanced, especially for genome-wide analyses. For this, we used the PRROC R package (https://cran.r-project.org/web/packages/PRROC).

## Additional file

Additional file 1 Additional figures and tables. **Figures S1–13** and **Tables S1, S2**. (PDF 1618 kb)
