# Supplementary material for: Predicting double-strand DNA breaks using epigenome marks or DNA at kilobase resolution
Source: Genome Biol. 2018 Mar 15;19:34. doi: 10.1186/s13059-018-1411-7 (PMC5856001; doi:10.1186/s13059-018-1411-7)
Supplement: Supplementary file 1 — Additional figures and tables. Figures S1–13 and Tables S1, S2. (PDF 1618 kb) [file 13059_2018_1411_MOESM1_ESM.pdf]

Additional File 1

Fig. S1 — Precision-recall (PR) curve of double-strand break prediction using epigenomic and chromatin data with random forest.

Area under the PR curve (AUPR) is plotted.

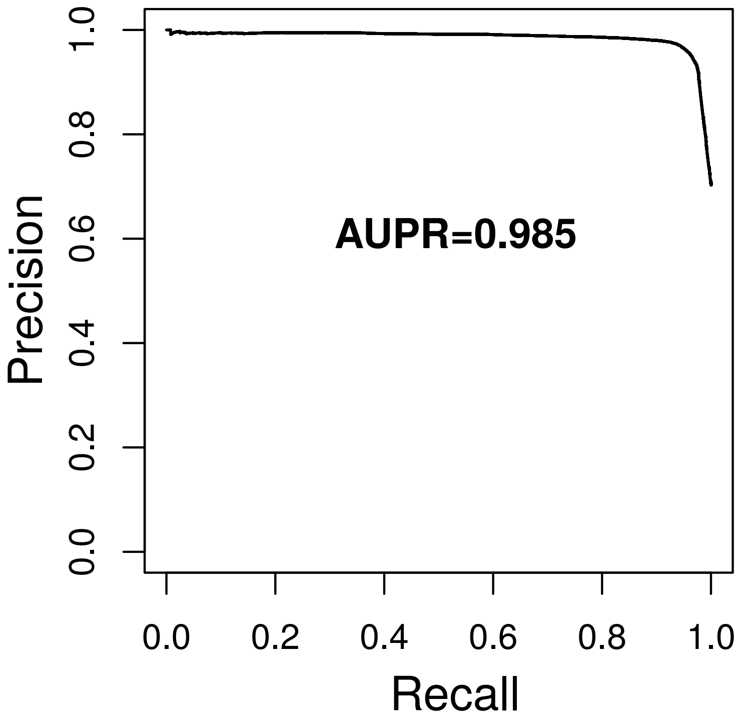

Fig. S2 — Prediction accuracy of double-strand breaks (DSBs) using epigenomic and chromatin data with lasso logistic regression.

a) Receiver operating characteristic (ROC) curve of the prediction of DSBs. Area under the ROC curve (AUROC) is plotted. b) Precision-recall (PR) curve. Area under the PR curve (AUPR) is plotted.

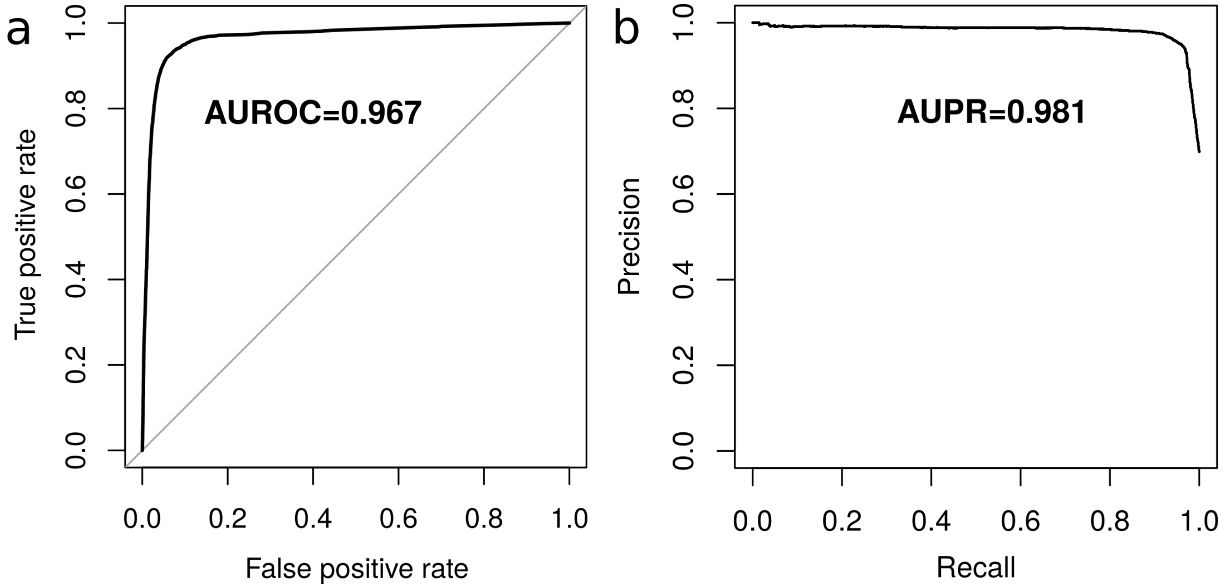

Fig. S3 — Comparison of lasso logistic regression coefficients with standard logistic regression coefficients for DSBCapture DSB prediction using epigenomic and chromatin data.

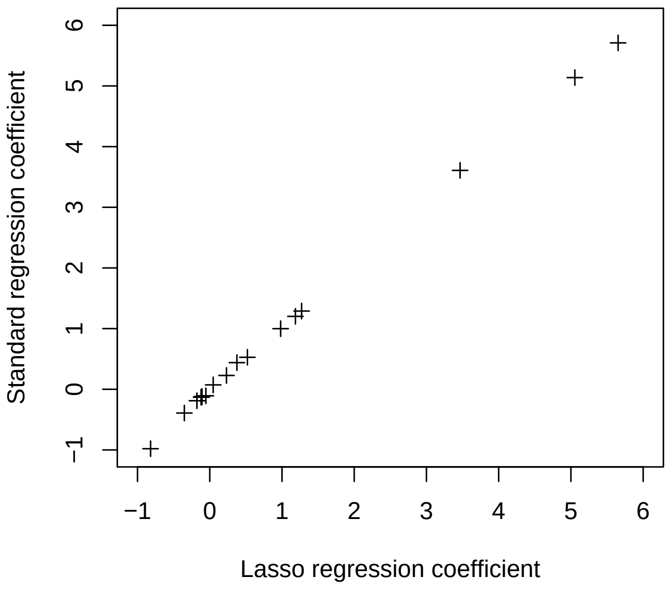

Fig. S4 — Precision-recall (PR) curve of double-strand break prediction using epigenomic and chromatin data with different random forest models.

Models include all variables, DNase only, H3K4me2 only, DNase+H2A.Z, or DNase+H3K4me1. Areas under the PR curves (AUPRs) are plotted.

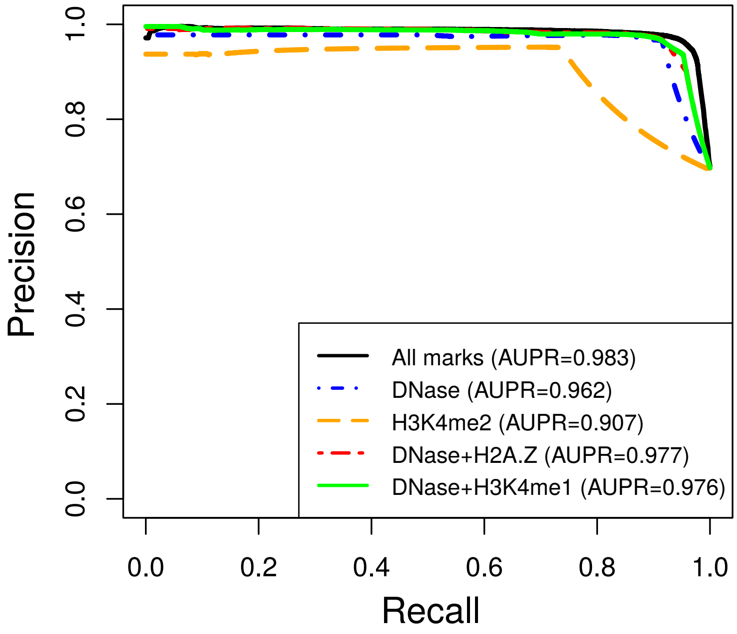

Fig. S5 — Prediction of double-strand breaks (DSBs) that did not overlap DNase sites using epigenomic and chromatin data with random forests.

a) Receiver operating characteristic (ROC) curve of the prediction of DSBs. Area under the ROC curve (AUROC) is plotted. b) Precision-recall (PR) curve. Area under the PR curve (AUPR) is plotted. c) Variable importances.

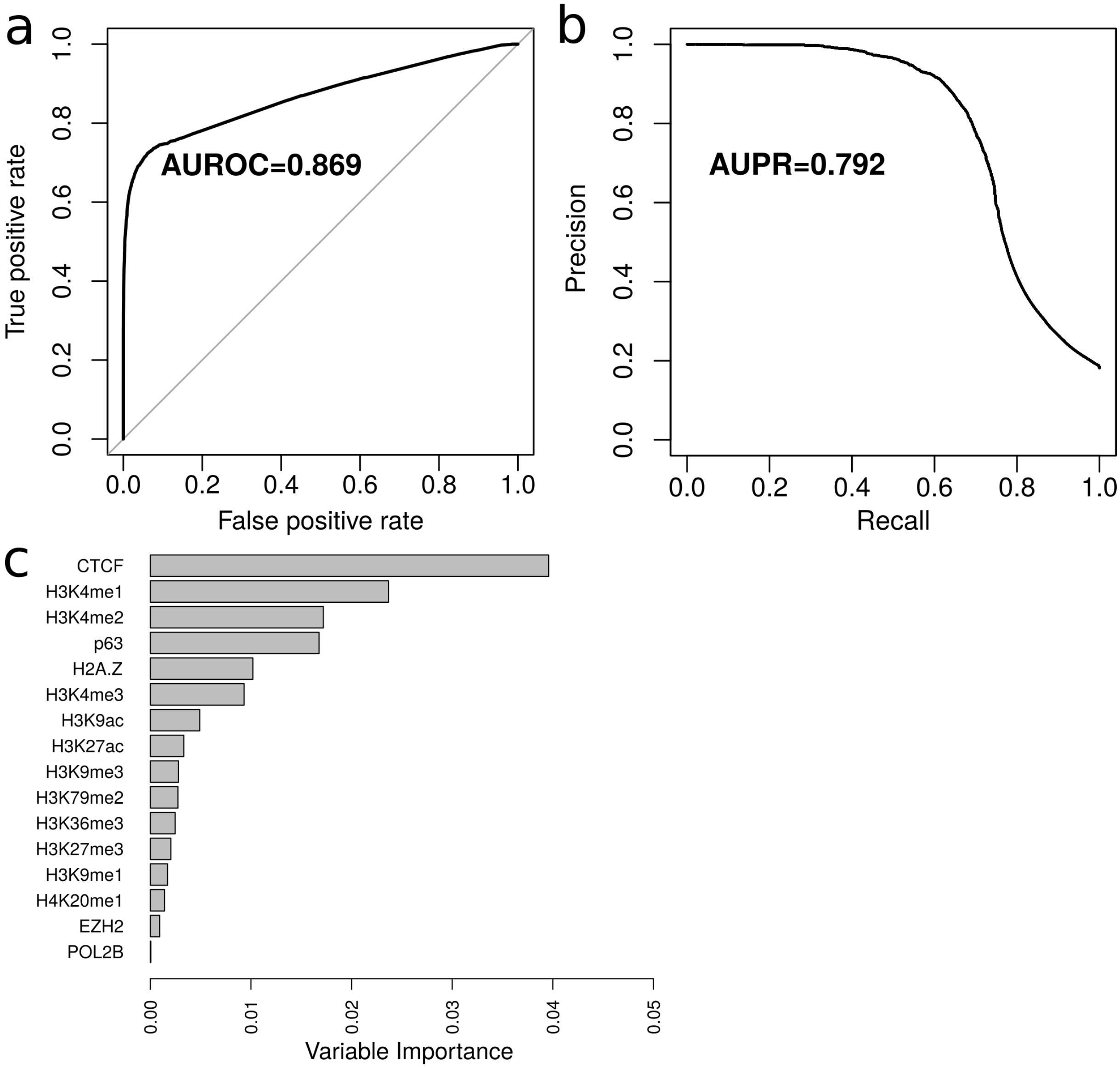

Fig. S6 — Comparison of precision-recall (PR) curves between DSBCapture-trained and BLESS-trained models.

Areas under the PR curves (AUPRs) are plotted.

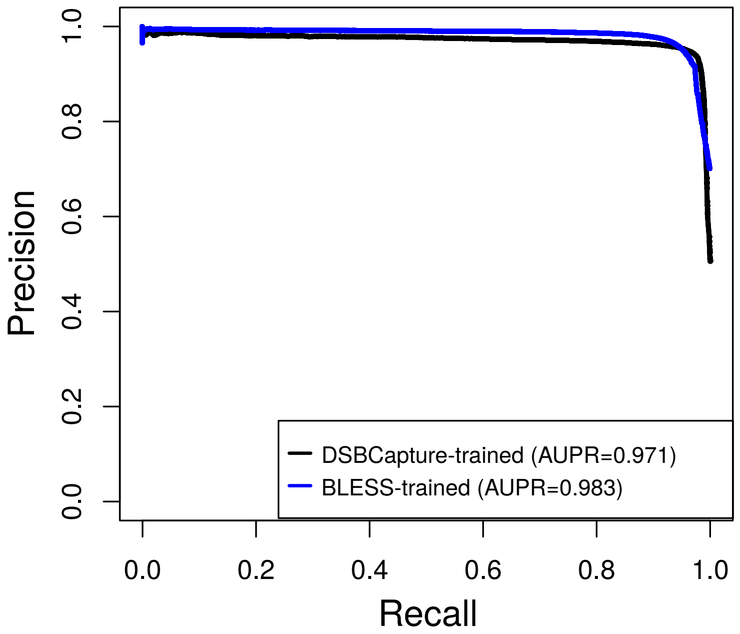

Fig. S7 — Precision-recall (PR) curves of double-strand break prediction using two different replicates.

Areas under the PR curves (AUPRs) are plotted. a) PR curve for the prediction of DSBs trained on replicate 1 and tested on same replicate. b) PR curve for the prediction of DSBs trained on replicate 1 and tested on replicate 2.

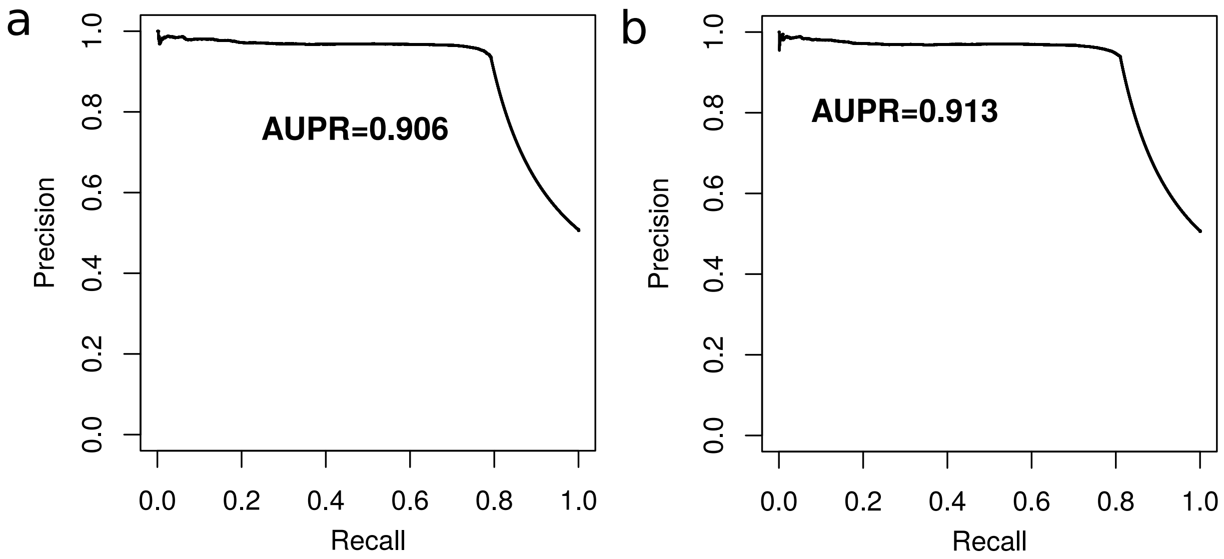

Fig. S8 — Illustration of double-strand break (DSB) prediction in another cell type.

a) Model training in NHEK cells. Only data that were available in both NHEK and U2OS cells were used: DNA-seq, CTCF, H3K4me1/3, H3K9me3, H3K27ac, H3K27me3, H3K36me3 and POL2B. b) Prediction of U2OS DSB sites using NHEK-trained model and U2OS ChIP-seq and DNase-seq data. c) Evaluation of U2OS DSB site predictions with U2OS DSBCapture, BLESS, XRCC4 and γ-H2AX data. Predictions were assessed using receiver operating characteristic (ROC) and precision-recall (PR) curves, and using enrichments of DSB features at predicted sites versus control sites.

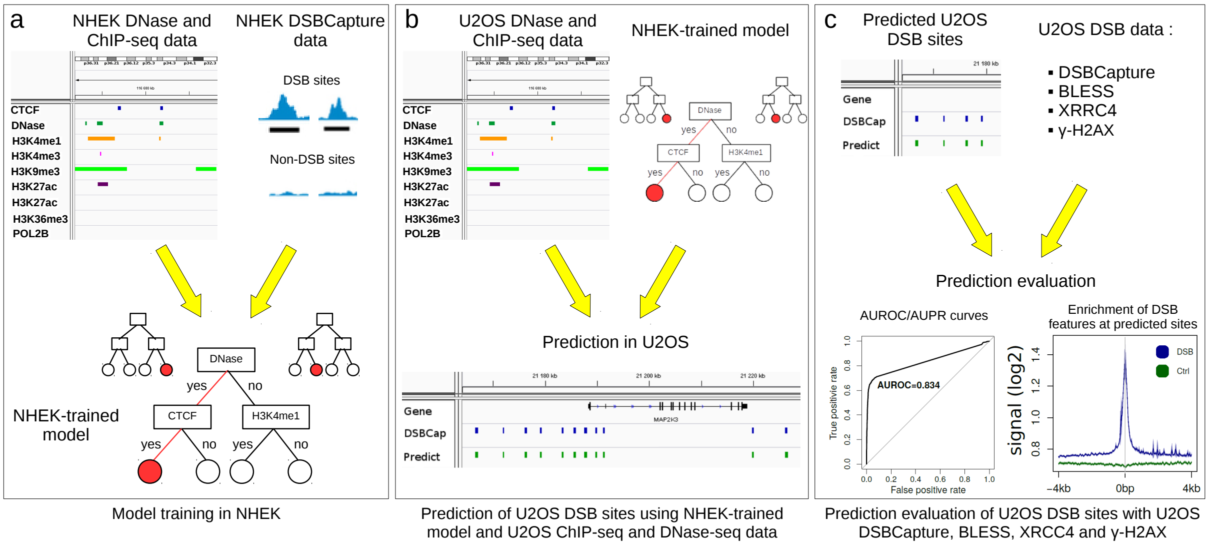

Fig. S9 — Precision-recall (PR) curve of the prediction of double-strand breaks using random forest learned from DSBs in one cell type (NHEK) to predict DSBs in another cell type (U2OS).

Areas under the PR curves (AUPRs) are plotted.

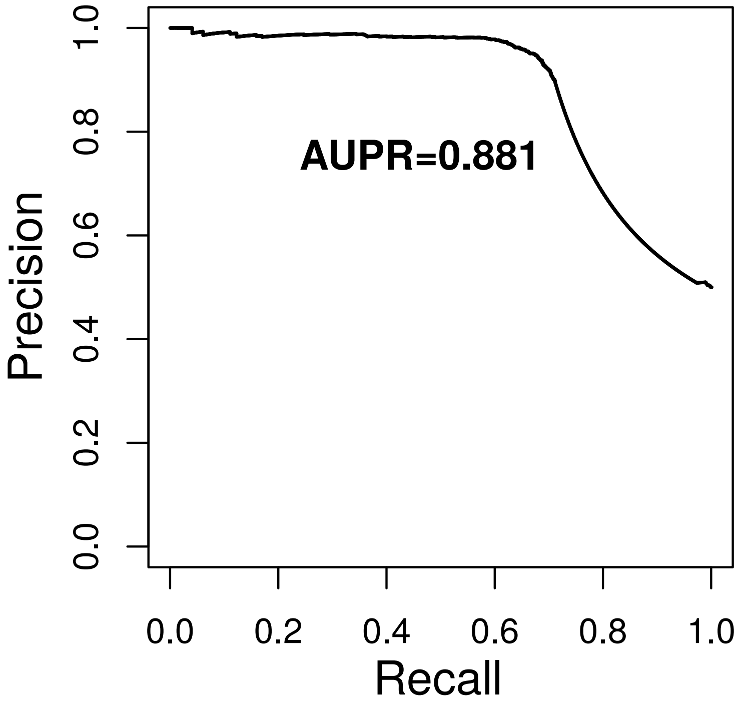

Fig. S10 — Prediction of double-strand breaks (DSBs) in U2OS using a random forest learned from U2OS DSB data.

a) Receiver operating characteristic (ROC) curve of the prediction of DSBs. Area under the ROC curve (AUROC) is plotted. b) Precision-recall (PR) curve. Area under the PR curve (AUPR) is plotted.

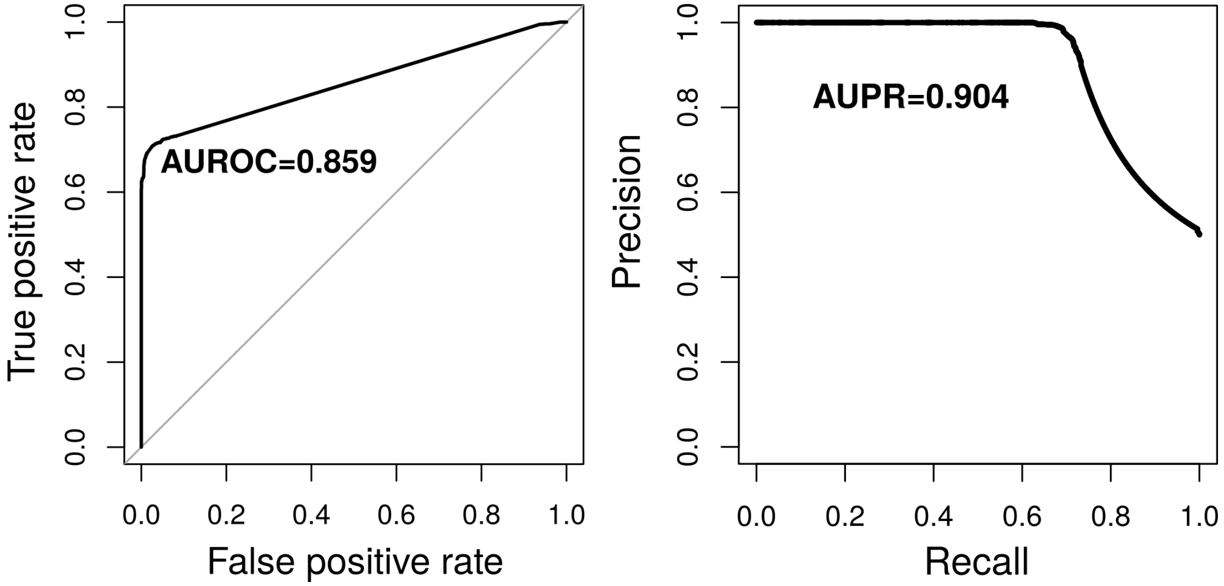

Fig. S11 — Average genome-wide predicted double-strand break (DSB) signal versus average signals of DSBCapture, BLESS, XRCC4 and γ-H2AX.

a) DSBCapture. b) BLESS. c) XRCC4. d) γ-H2AX. DSBs were predicted for all 250 base bins of the genome, then grouped into 10 classes depending on the predicted DSB signal: [0-0.1[, [0.1-0.2[, [0.2-0.3[, [0.3-0.4[, [0.4-0.5[, [0.5-0.6[, [0.6-0.7[, [0.7-0.8[, [0.8-0.9[and [0.9-1]. For each class, DSBCapture, BLESS, XRCC4 and γ-H2AX signals were averaged and plotted.

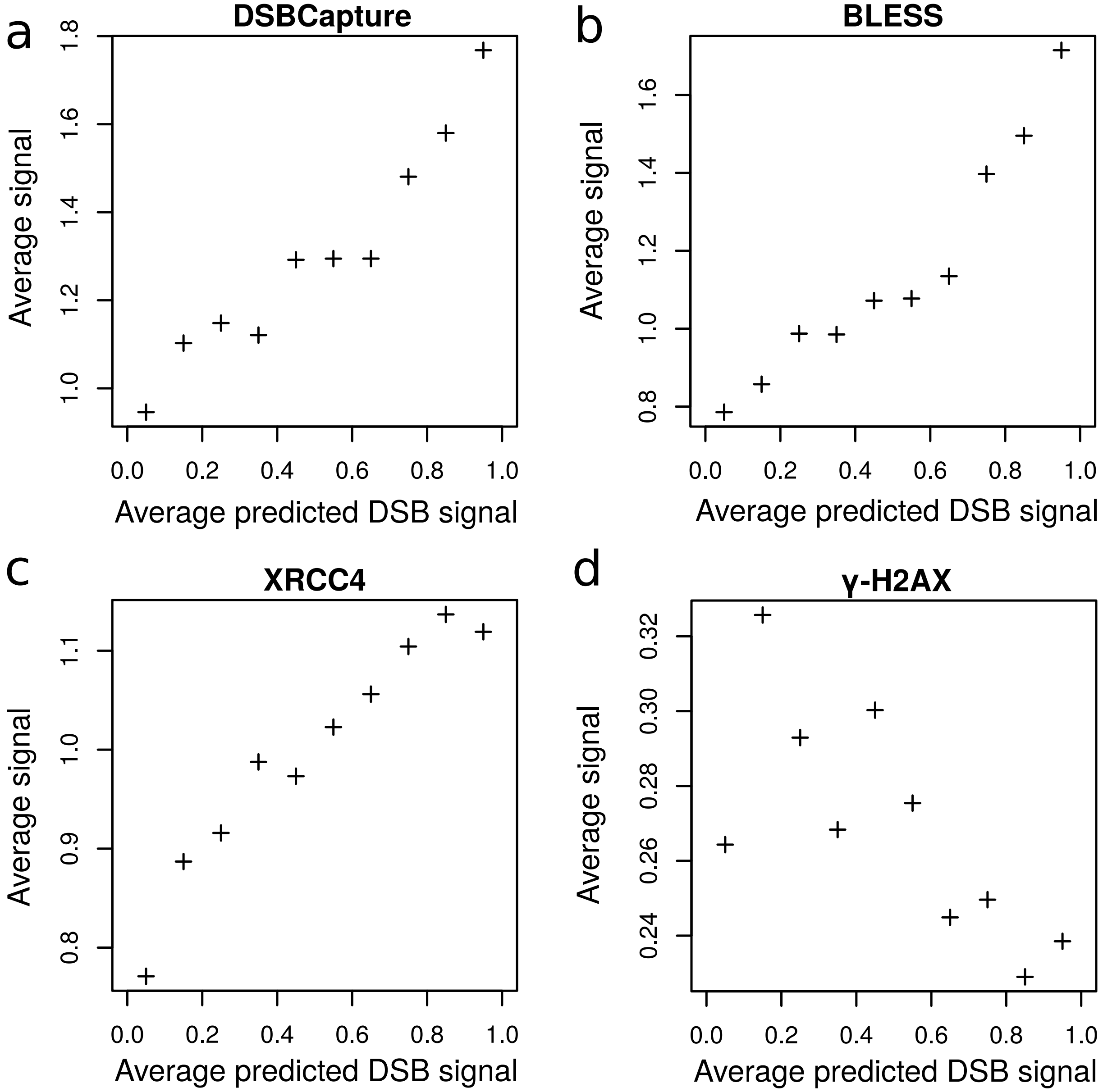

Fig. S12 — Prediction of double-strand breaks (DSBs) in KBM7 and MCF-7 cells using a random forest learned from NHEK DSB data.

a) Average profile of KBM7 BLISS at predicted KBM7 DSB regions compared to non-DSB regions over the whole genome. b) Average genome-wide predicted KBM7 double-strand break (DSB) signal versus average signals of KBM7 BLISS. DSBs were predicted for all 250 base bins of the genome, then grouped into 10 classes depending on the predicted DSB signal: [0-0.1[, [0.1-0.2[, [0.2-0.3[, [0.3-0.4[, [0.4-0.5[, [0.5-0.6[, [0.6-0.7[, [0.7-0.8[, [0.8-0.9[and [0.9-1]. For each class, BLISS signals were averaged and plotted. c) Average profile of MCF-7 END-seq at predicted MCF-7 DSB regions compared to non-DSB regions over the whole genome. d) Average genome-wide predicted MCF-7 double-strand break (DSB) signal versus average signals of MCF-7 END-seq. e) Average profile of etoposide (ETO) treated MCF-7 END-seq at predicted MCF-7 DSB regions compared to non-DSB regions over the whole genome. f) Average genome-wide predicted MCF-7 double-strand break (DSB) signal versus average signals of etoposide treated MCF-7 END-seq.

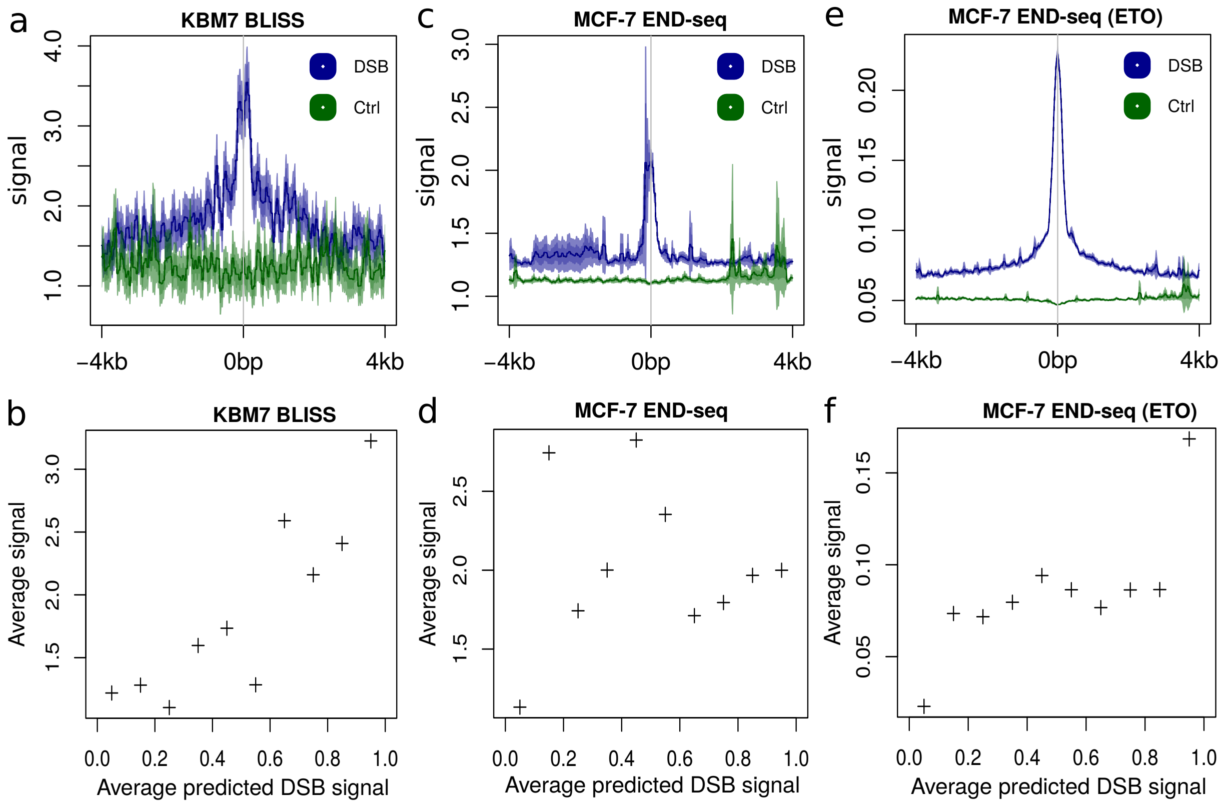

Fig. S13 — Prediction of double-strand breaks (DSBs) using DNA motifs and shape.

a) Precision-recall (PR) curve of the prediction using DNA motifs only. b) PR curve of the prediction using DNA motifs and shape. Areas under the PR curves (AUPRs) are plotted.

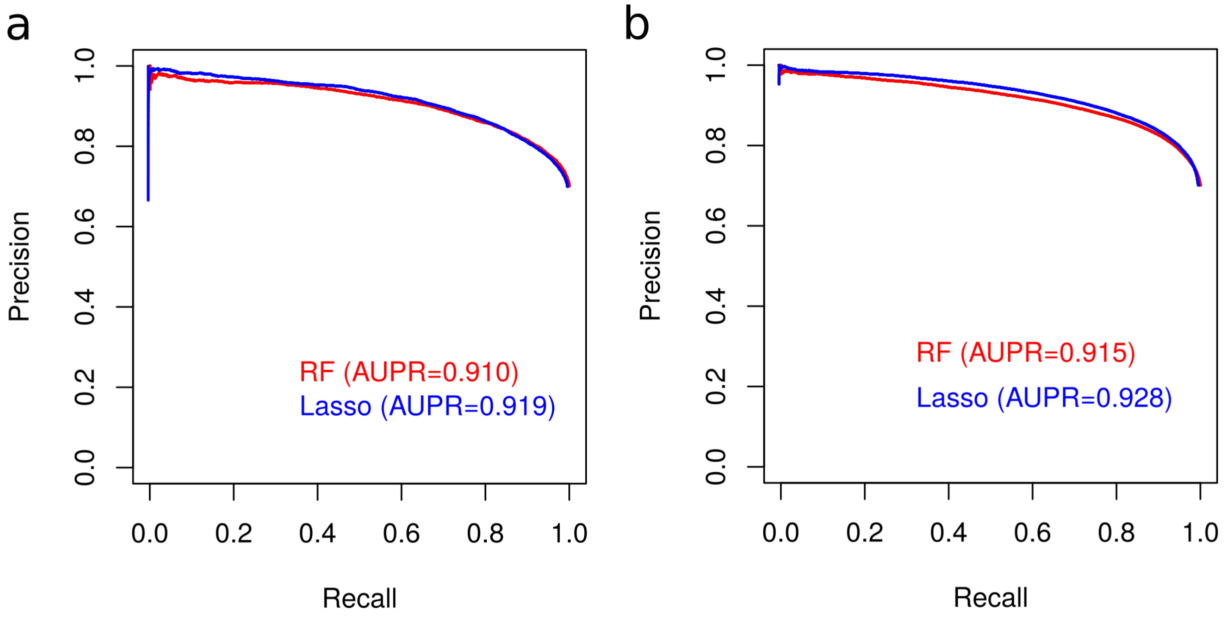

Table S1 — Standard logistic regression coefficients with p-values for DSB-Capture DSB prediction using ChIP-seq and DNase-seq data.

Variable	Coefficient	Standard Error	Z	p value
Intercept	-2.09	0.03	-83.24	0
CTCF	5.14	0.11	46.60	0
Dnase	5.71	0.06	96.36	0
EZH2	1.29	0.21	6.01	1.80903E-09
H2A.Z	0.53	0.03	15.31	7.00231E-53
H3K27ac	-0.39	0.06	-7.06	1.65769E-12
H3K27me3	0.23	0.03	6.54	6.07165E-11
H3K36me3	-0.19	0.04	-4.55	5.3508E-06
H3K4me1	1.20	0.04	31.45	3.8729E-217
H3K4me2	1.00	0.05	19.67	4.26257E-86
H3K4me3	0.44	0.06	7.17	7.75307E-13
H3K79me2	-0.13	0.05	-2.71	0.006735905
H3K9ac	-0.11	0.06	-1.79	0.073940384
H3K9me1	-0.13	0.03	-3.81	0.000140859
H3K9me3	-0.12	0.04	-3.21	0.001338959
H4K20me1	0.07	0.04	1.99	0.046352195
p63	3.61	0.14	25.20	4.373E-140
POL2B	-0.98	0.20	-4.91	9.24534E-07

Table S2 — Standard logistic regression coefficients with p-values for DSB-Capture DSB prediction using DNA motif occurrence data.

Only motifs with lasso coefficients larger than 0.5 were used in this regression.

Motif	Coefficient	Standard Error	Z	p value	FDR corrected P value
(Intercept)	0.52	0.01	69.48	0	0
ATF4_MA0833.1	1.11	0.20	5.67	1.40645E-08	0.000000051
CTCF_MA0139.1	3.75	0.25	14.91	2.76752E-50	2.0064547E-49
ESR1_MA0112.2	11.55	195.41	0.06	0.952853398	0.9725154782
ESR1_MA0112.3	-0.20	0.84	-0.24	0.81137307	0.9245077019
ESR2_MA0258.2	0.64	0.19	3.36	0.000790845	0.001349089
FOSL1_MA0477.1	1.10	0.04	24.91	5.2765E-137	7.650867E-136
FOS_MA0476.1	1.37	0.04	34.81	1.551E-265	4.497881E-264
GLI2_MA0734.1	1.21	0.34	3.54	0.000397598	0.0007686902
HINFP_MA0131.2	2.16	0.22	9.63	6.1566E-22	2.9756922E-21
IRF2_MA0051.1	0.96	0.49	1.95	0.050925544	0.0615350321
JUN_MA0488.1	0.42	0.08	5.01	0.000000553	1.2335855E-06
MTF1_MA0863.1	13.04	378.59	0.03	0.972515478	0.9725154782
NFYA_MA0060.1	1.27	0.09	14.50	1.14146E-47	6.620458E-47
NR3C1_MA0113.3	0.99	0.42	2.39	0.016787761	0.0221293215
NRF1_MA0506.1	2.13	0.11	19.09	3.34881E-81	3.2371801E-80
REST_MA0138.1	1.37	0.43	3.15	0.001629588	0.0023629033
REST_MA0138.2	1.97	0.56	3.52	0.00043294	0.0007847031
RFX4_MA0799.1	1.56	0.32	4.92	8.69584E-07	1.8012815E-06
SMAD3_MA0795.1	0.56	0.17	3.31	0.000938939	0.0015015814
SRF_MA0083.1	0.68	0.28	2.46	0.013823085	0.0190890225
TFCP2_MA0145.3	0.49	0.10	5.08	3.77948E-07	9.1337443E-07
TP53_MA0106.2	1.49	0.27	5.41	6.13873E-08	0.000000178
TP63_MA0525.1	2.36	0.36	6.49	8.52911E-11	3.5334869E-10
TP63_MA0525.2	11.00	192.26	0.06	0.954393626	0.9725154782
TP73_MA0861.1	11.73	54.25	0.22	0.828868974	0.9245077019
XBP1_MA0844.1	1.25	0.38	3.30	0.000983795	0.0015015814
ZBTB33_MA0527.1	2.12	0.38	5.62	1.87922E-08	6.0552682E-08
ZBTB7A_MA0750.1	1.67	0.32	5.30	1.18616E-07	3.1271509E-07
ZNF143_MA0088.2	1.51	0.75	2.01	0.044143699	0.0556594465
